# Reduction of the Number of Samples for Cost-Effective Hyperspectral Grape Quality Predictive Models

**DOI:** 10.3390/foods10020233

**Published:** 2021-01-23

**Authors:** Julio Nogales-Bueno, Francisco José Rodríguez-Pulido, Berta Baca-Bocanegra, Dolores Pérez-Marin, Francisco José Heredia, Ana Garrido-Varo, José Miguel Hernández-Hierro

**Affiliations:** 1 Food Colour and Quality Laboratory, Área de Nutrición y Bromatología, Facultad de Farmacia, Universidad de Sevilla, 41012 Sevilla, Spain; julionogales@us.es (J.N.-B.); rpulido@us.es (F.J.R.-P.); heredia@us.es (F.J.H.); jmhhierro@us.es (J.M.H.-H.); 2 Department of Animal Production, Campus de Rabanales, Universidad de Córdoba, 14071 Córdoba, Spain; dcperez@uco.es (D.P.-M.); pa1gavaa@uco.es (A.G.-V.)

**Keywords:** hyperspectral imaging, near-infrared, grape quality, sample selection, chemometrics

## Abstract

Developing chemometric models from near-infrared (NIR) spectra requires the use of a representative calibration set of the entire population. Therefore, generally, the calibration procedure requires a large number of resources. For that reason, there is a great interest in identifying the most spectrally representative samples within a large population set. In this study, principal component and hierarchical clustering analyses have been compared for their ability to provide different representative calibration sets. The calibration sets generated have been used to control the technological maturity of grapes and total phenolic compounds of grape skins in red and white cultivars. Finally, the accuracy and precision of the models obtained with these calibration sets resulted from the application of the selection algorithms studied have been compared with each other and with the whole set of samples using an external validation set. Most of the standard errors of prediction (SEP) in external validation obtained from the reduced data sets were not significantly different from those obtained using the whole data set. Moreover, sample subsets resulting from hierarchical clustering analysis appear to produce slightly better results.

## 1. Introduction

Near-infrared spectroscopy (NIRS) is applied today to monitor a large number of parameters in the food sector. Traditional spectroscopy provides useful methods that are applied continuously in both food research and the food industry [1,2,3]. Particularly, in the sector of viticulture, NIRS has been increasingly applied to grape quality assessment as a rapid and non-destructive technique. NIRS can measure the absorption of electromagnetic radiation at wavelengths in the range 780–2500 nm. The NIR spectra of grapes (as well as other food products) comprise broad bands arising from overlapping absorptions corresponding mainly to overtones and combinations of vibrational modes, involving C–H, O–H, and N–H chemical bonds. This makes NIRS a very feasible tool to measure organic and biological systems such as grape samples [4]. NIRS has strongly demonstrated its suitability for on-site and real-time quality control at different points of the wine production chain. This technique can be applied to monitor grape quality during on-vine ripening, helping, in this way, in the decision-making process. In addition, the implementation of NIRS methods allows us to improve the sampling procedure, which translates into better use of resources and a greater capacity for analysis [5,6,7]. Three comprehensive reviews show the potential and challenges of NIRS for analysis of the chemical composition of grapes in the laboratory, the vineyard and before or during the harvest, to provide better insights into the chemistry, nutrition and physiology of grapes [5,8,9].

In the last decade, image analysis has been added to spectroscopy resulting in hyperspectral imaging. The inclusion of the spatial domain allows some quantitative and qualitative approximations that are not possible with traditional single-point spectroscopy [10,11]. Several hyperspectral studies have been carried out, in our laboratory, for the analysis of the chemical composition of grapes [12], grape maturity [13] and to measure phenols concentration in grape or grape seeds [14,15]. Other authors have used near-infrared hyperspectral imaging to quantify these quality parameters in the lab [16,17] or even in the field [18].

Both traditional NIR spectroscopy and hyperspectral image analysis require the use of a representative calibration set of the entire population. Therefore, generally, the calibration procedure requires a large number of resources, not only in the spectra acquisition step but also in the determination of the reference parameters [19]. Some of the reference analyses used in the oenological sector are based on time-consuming and polluting methodologies. Chromatographic or spectrophotometric methods are usually applied to the determination of reference parameters such as the contents of total phenols, individual phenolic compounds, total acidity, elemental composition, etc. [20,21]. For that reason, in a real-world situation, the number of samples that can be used for developing a regression model is usually small due to budget and/or time constraints [22]. Thus, the optimal sample size is often determined by a balance between the available budget and acceptable accuracy. Furthermore, the calibration sampling strategy is crucial when the number of samples that can be included in the calibration set is restricted. It exists a relation between the calibration sampling strategy and the generalization ability of the models [22,23]. Therefore, there is a great interest in identifying the most representative samples within a complete set of samples to reduce the number of resources required without losing information that could be important for the development of chemometric models.

A good option to achieve a representative subset of the spectral space is to study the spectral distribution of the samples in that space and to take into account this distribution in the selection procedure. To do that, the spectral distances between samples and the population center are usually measured. Euclidean and Mahalanobis distances are employed to evaluate the distribution of the spectra in a spectral space [24]. Shenk and Westerhaus [25,26] patented the algorithms denominated CENTER and SELECT, based on measuring the Mahalanobis distance (H) and Neighbourhood Mahalanobis distance (NH). These algorithms allow to structure spectra within a spectral matrix and to select the most representative spectra for their subsequent analysis. When H is calculated using a small number of latent variables, i.e., principal components (PCs) obtained after a principal component analysis (PCA), some problems, such as multicollinearity are avoided [24]. The global H measures the distance of each sample to the center of the sample population. Samples with an H value greater than 3 are considered spectral outliers [26]. The NH calculates the distance between pairs of samples. An NH value lower than 0.6 indicates that the two spectra are similar to each other (‘neighbor’). These algorithms have been extensively used with NIRS data to study the structure and variability of the sample population and to select samples, based on their spectral features, for several applications, such as calibration development or spectral instrument matching applications [27,28].

In addition, different multivariate strategies can be applied, based on the measurement of other spectral distances or differences between samples (k-nearest neighbors, Kennard-Stone, successive projections algorithm, etc.). For example, Kennard-Stone (KS) algorithm [29], a classic method for sample selection, calculates the distance between samples, selecting samples uniformly distributed in the predictor space. KS commonly uses the Euclidean distance and has been widely applied to select spectral samples in agricultural and food products such as soy sauce [30], corn gluten meal [31] and grasses [32], among others. Other statistical tools, such as dendrograms or clusters analysis, have also been applied to identify representative samples within a spectral dataset. For instance, Moros et al. [33] made up their calibration and validation sets from a dendrogram obtained after hierarchical cluster analysis of NIR spectra of soils. Using this sample selection, they developed prediction models for the screening of physicochemical parameters of soil samples obtaining similar or lower errors than those of the models reported in literature developed without sample selection.

To check what is the better sample selection method for identifying representative grape samples according to their NIR spectra, a comprehensive study has to be developed. Consequently, the main aim of this study was to check the feasibility of different sample selection methods for making up representative sample sets of grape spectra. Then, calibration models for the prediction of total acidity, total soluble solids, total skin phenols and pH were developed for the entire sample set and the different representative calibration sets selected and, finally, results were compared.

## 2. Materials and Methods

The grape samples used, their spectral and chemical information acquisition and some of the chemometric methods applied in this study were deeply described by Nogales-Bueno, Hernández-Hierro, Rodríguez-Pulido and Heredia [13]. However, the present study describes a new and different approach based on these data and, therefore, they are briefly described in the following sections. Moreover, sample selection procedures are described in detail.

### 2.1. Samples

A total of 213 grape samples (*Vitis vinifera* L.) were collected from 4 different vineyards located in the Condado de Huelva Designation of Origin D.O. (Andalusia, Spain) at different dates from mid-July to early September during the 2012 vintage. Samples belonged to Syrah and Tempranillo red varieties and Zalema white variety. Samples were collected weekly since the pre-bloom period to the vintage of each vineyard. In that way, different stages of maturity were taken into account. From each vineyard and date, at least 1.5 kg of grapes were collected. With the aim of achieving representative samples, they were collected from several rows of vines distributed homogeneously in each vineyard. In these rows, grapes were collected from the top, middle and bottom of the cluster, and in the sunlight and shade side of this. Then, samples were carried to the laboratory, where a subgroup of 20–30 berries was randomly selected for each sample. Later on, their spectra were acquired and their reference composition was determined.

### 2.2. Spectral Matrix

A hyperspectral image of each sample was recorded, comprising 20 to 30 berries each. Spectral images were acquired with a pushbroom hyperspectral device (Infaimon S.L., Barcelona, Spain). This device comprised a Xenics^®^ XEVA-USB camera (320 × 256 pixels; Xenics Infrared Solutions, Inc., Leuven, Belgium) with an InGaAs sensor covering the spectral range between 900 and 1700 nm. Samples were placed at the bottom of the device and two halogen lamps illuminated them at a 45° angle to avoid specular reflection and maximize the scattering effects. Spectral images were saved in matrix files with two spatial and one spectral dimension. In each acquisition session, the spectral information of an almost totally reflective tile and the dark current of the camera was acquired. With this information, the spectra of the samples were calibrated and then corrected images were saved.

Hyperspectral images were segmented to identify the background and the sample pixels applying stepwise linear discriminant analysis. A discrimination function was constructed using the reflectance values of these six wavelengths (979, 1034, 1073, 1314, 1386 and 1550 nm) retained by the discriminant analysis. After that, only the sample pixels were taken into account and the average spectrum of each sample was used for the development of the rest of the study. Two spectra of white and red grape samples are included as Supplementary Material (Figure S1).

### 2.3. Reference Parameters

The number of reference parameters were determined and were used to evaluate the goodness of the different sample sets constructed. Reference parameters were selected for being useful and widely employed in the oenological industry. They are usually employed for controlling grape quality and establishing the grape harvest time. For each sample, total acidity, total soluble solids, total skin phenols and pH were determined. For the determination of total acidity, total soluble solids and pH, grape must was obtained. The total acidity of the must is the sum of its titrable acidities when it is titrated at pH = 7 against a standard alkaline solution. Soluble solids were obtained by densimetry and pH was directly measured in the must. Total phenols of grape skins were determined using the Folin–Ciocalteu method [34]. For that, grape skins were macerated in acidified methanol. Later, methanolic supernatants were evaporated and redissolved in water. This solution was subjected to the spectrophotometric method and total phenols were obtained as gallic acid equivalents per gram of grape skin. All methods used for the determination of the reference parameters are recommended by the Organisation Internationale de la Vigne et du Vin (OIV) [20].

### 2.4. Sample Selections

From the whole set of samples, one-third of them (71 samples) were randomly selected to build an external validation set. This validation set was saved and later used to develop external validations in all the calibration models developed in this study. The remaining two-thirds of samples (142 samples), in the following the full calibration (FC) set, were used for building different calibration sets.

In the first step, a principal component analysis (PCA) was applied to the spectra comprised the FC set to look for possible spectral outliers and to sort the samples according to their spectral variability. The PCA explains 99% of the spectral variability of the FC set using 15 principal components (PCs) [13]. The information provided by the PCA was used to more easily achieve the selection of representative sample sets. Two different methods were applied for obtaining representative sample selections: Neighbourhood Mahalanobis (NH) distance and hierarchical clustering analysis (HC).

Mahalanobis distances (H) were measured between all samples and the average spectra of the FC set. Samples with an H value higher than 3 were identified as spectral outliers and deleted from that set. Following that procedure, one spectral outlier was identified and eliminated. This sample of Zalema variety was no longer considered in the rest of the study. Moreover, H distances were also used to calculate the NH distances between samples. Then, samples were grouped according to their NH distance and these groups were used for selecting spectrally representative calibration sets (in the following the NH sets) [35]. PCA and NH selection were performed using Win ISI^®^ (v1.50) (Infrasoft International, LLC, Port. Matilda, PA, USA).

On the other hand, the PCA scores were submitted to a hierarchical clustering analysis. Hierarchical clustering is a general approach to cluster analysis, in which objects are analyzed to look for their similarities, therefore, it is a potent tool for grouping spectra and selecting the most representative [33]. In the present study, a divisive process based on the squared Euclidean distances and Ward linkage method was used. Ward’s method uses an analysis of variance approach to evaluate the distances between clusters. In short, this method attempts to minimize the sum of squares of any two (hypothetical) clusters that can be formed at each step [36]. Then, the graphical representation of the hierarchical clustering analysis, or dendrogram, was constructed. Selecting different Ward linkage distances, samples were divided into different homogenous groups and these groups were used for selecting spectrally representative calibration sets (in the following the HC sets). Hierarchical clustering analysis and dendrograms were calculated using Statistica v.8.0 software (StatSoft Inc., Tulsa, OK, USA).

### 2.5. Modified Partial Least Square (MPLS) Regressions on the Full Calibration (FC) Set

Initially, the FC set was used to establish the potential of this sample set for the prediction of the reference parameters. For that, an Modified Partial Least Square (MPLS) regression was developed for each reference parameter. This procedure is broadly described by Nogales-Bueno, Hernández-Hierro, Rodríguez-Pulido and Heredia [13]. However, since the results of this study were used to evaluate the goodness of the different sample selections developed in this study, a brief summary is included. For calibration optimizing, different signal pretreatments were applied to the spectra. Standard normal variate (SNV), multiplicative scattering correction (MSC), detrend and different derivatives were tested, as is described in detail in [14]. MPLS regressions were developed for each reference parameter and standard errors of cross-validation (SECV) were evaluated. In this method, the group of calibration samples is divided into a series of subsets to perform cross-validation to set the number of Partial Least Square (PLS) factors, reducing the possibility of overfitting [37]. Chemical outliers were identified using the critical T outlier value. These chemical outliers were removed applying the T ≥ 2.5 criterion, i.e., eliminating samples that presented a high residual value when they were predicted. Only the model with the lowest SECV was saved for each reference parameter. Then, samples belonging to the external validation set were predicted and standard errors of prediction (SEP) were obtained. The most effective pretreatments were MSC for total acidity, MSC plus first derivative for total soluble solids, SNV plus second derivative for total skin phenols and MSC plus second derivative for pH. The obtained models presented a good potential for a fast and reasonably inexpensive screening of these parameters [13].

Afterwards, similar MPLS regressions were developed for the NH and HC calibration sets (i.e., the sample sets confirmed after the different sample selections). For each reference parameter, the same pretreatment that produced the best results for the FC set was applied. Then, SECV and SEP were evaluated and SEP were compared to those obtained without sample selection (FC set) using a Fisher test [38,39], as described in detail in Pérez-Marín et al. [40].

## 3. Results and Discussion

### 3.1. Sample Selection Using Neighbourhood Mahalanobis (NH) Distance

Initially, NH = 0.6 threshold was set. This threshold is the most frequently applied in the available literature [26,27,41], although in most cases, it is applied to matrices really different from the grape. Particularly, these examples were forages, commercial feeds and oak wood shavings, samples with different structures and compositions than grapes. However, due to its widespread use, this threshold value is a logical starting point for assessing the relationship between spectral distance and actual differences in grape samples. After obtaining all the NH distances between samples, 79 groups of spectrally homogeneous samples were created. By selecting one sample from each group, an NH calibration set of 79 samples was obtained. This number of samples represent most of the 55% of the samples present in the FC set. To reduce the number of selected samples, a higher NH was tested. The higher the NH threshold, the bigger the size of the groups and, in consequence, the lower the number of selected samples. Choosing an NH threshold of 0.9, as is also applied in some studies [26,42], the number of groups obtained was 42, i.e., almost 30% of the samples allocated in the FC set.

**Figure 1:**
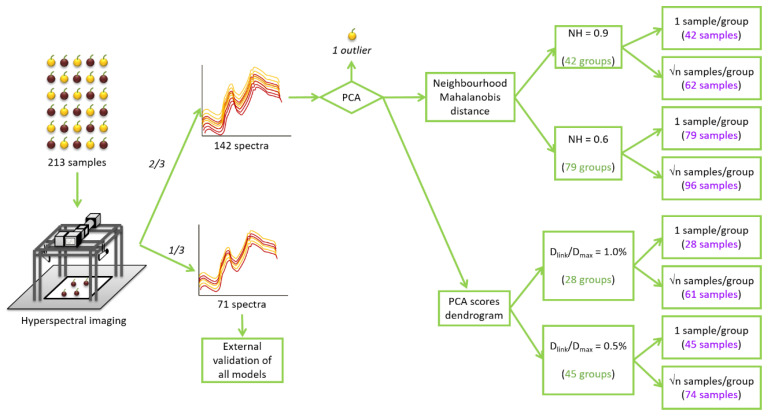
Schematic representation of the spectra acquisition and the sample selection procedure. NH: Neighbourhood Mahalanobis distance; Dlink: different linkage distances; Dmax: the maximum linkage distance.

The described algorithm produces groups with a different number of samples and it automatically selects the most central sample of each group. For example, for NH = 0.9, the number of samples per group ranged from 1 to 30 samples. Therefore, selecting only one central sample per group can be adequate in groups with a reduced number of samples, but insufficient for the bigger groups. To solve this issue, another sample selection methodology was also applied: selecting √n amples (square root of the number of samples in a group) per group [43,44]. In this case, taking into account the NH distance between each sample and the central one, the samples were selected to be as well distributed as possible in the group. This methodology increased the number of samples to 62 and 96 samples for both thresholds of 0.9 and 0.6, respectively. In consequence, four NH calibration sets were obtained using the NH sample selection procedure (Figure 1).

### 3.2. Sample Selection using Hierarchical Clustering (HC) Analysis

**Figure 2:**
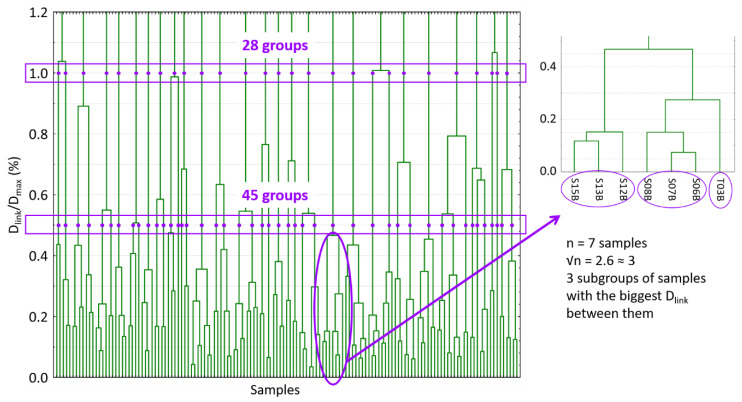
Dendrogram produced in the hierarchical clustering analysis. The two linkage distances and the different groups produced are marked. Moreover, the procedure of selecting √n samples of one group is also shown as an example. D_link_: different linkage distances; D_max_: the maximum linkage distance; √n:square root of the number of samples in a group.

Scores of the first 15 PCs of the PCA analysis were used for the development of hierarchical clustering analysis. Squared Euclidean distances were calculated and the Ward linkage method was applied to order and split in different groups the spectral samples allocated into the FC set. In that way, a dendrogram was constructed (Figure 2).

The hierarchical clustering analysis links samples according to the distance between them. It established the maximum linkage distance (D_max_) between samples at 3 and, then, it represents the different linkage distances (D_link_) as a percentage of that maximum distance. In Figure 2, the number of created groups depends on the linkage distance selected. As can be seen, for values of D_link_/D_max_ of 1.0% and 0.5%, they can be identified 28 and 45 different groups of samples, respectively. In that way, two different HC set of samples can be constructed by randomly selecting one sample from each group. Furthermore, in order to obtain two more sample sets and to take into account the different sizes of the groups, √n samples per group were selected per each linkage distance. In this case, groups were divided into √n subgroups (the nearest whole number). Subgroups were constructed with the largest D_link_/D_max_ between them (Figure 2). One sample was randomly chosen for each subgroup. Therefore, 4 HC calibration sets were obtained with 28, 45, 61 and 74 samples (Figure 1).

### 3.3. Modified Partial Least Square (MPLS) Regressions on the NH and HC Sets

Following the procedure described above, MPLS regressions were applied to the 4 NH and 4 HC sample sets. The corresponding reference parameter values were added to each spectrum of these calibration sets. Reference parameters were used as dependent (Y) variables, whereas, the different wavelengths in the grape spectra were used as independent (X) variables. MPLS calibrations were obtained for the prediction of total acidity, total soluble solids, total skin phenols and pH. SECV values were obtained after the cross-validation procedure. The statistical parameters of the different calibrations are shown in Table 1.

To evaluate the results of the different models obtained, their statistical parameters were compared with those obtained from the FC set and described by Nogales-Bueno, Hernández-Hierro, Rodríguez-Pulido and Heredia [13]. These results are also included in Table 1 for easier interpretation. Almost all models developed from NH sets showed similar accuracy to FC models. For example, similar SECV values were obtained for total soluble solids and pH using the NH set constructed with NH = 0.6 and choosing √n sample per group. In that case, SECV obtained is lower or similar to those obtained with the FC set. Taking into account the HC sample sets, those built with a D_link_/D_max_ of 0.5 and choosing √n samples per group show the best results. These models, calculated from 74 samples (52% of the FC sample set), show slightly higher SECV values than those obtained with FC sets for all reference parameters.

**Figure 3:**
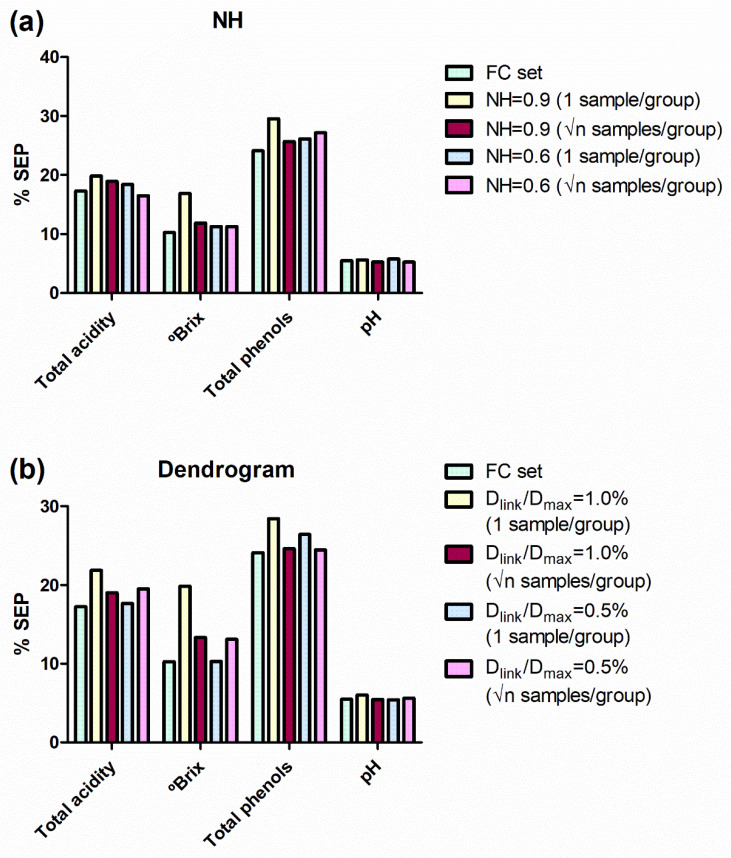
SEP in external validation for the models developed for all the reference parameters and for all the sample sets. SEP values are expressed as percentages taking into account the applicability range of their respective models. (**a**) Comparison for NH sample selection. (**b**) Comparison for Hierarchical Clustering (dendrogram) sample selection. SEP: standard errors of prediction; NH: Neighbourhood Mahalanobis distance; FC, full calibration; D_link_: different linkage distances; D_max_: the maximum linkage distance; √n: square root of the number of samples in a group.

However, to confirm which sample selection procedure enables to get better sample selection and, therefore, better MPLS models, external validation was performed. The external validation set, initially reserved for this purpose, was used (Figure 1). The SEPs obtained using the different sample selection sets were compared to those obtained without sample selection (FC set) using Fisher’s test, to determine whether differences between them were statistically significant (α = 0.05%). For each reference parameter, almost all SEP values obtained using the NH and HC sets were not statistically different from those obtained using the FC set. Only two models, developed for total soluble solids (one NH and one HC), showed SEPs significantly higher than the FC model (Table 1). In order to easily interpret these results, they were transformed to percentages taking into account the applicability range of their respective models and compared with errors in the FC set (Figure 3).

In general, the SEP values obtained from NH and HC sets are similar to those obtained from the FC set. For NH sets, the SEP values were similar to those obtained for the FC set (Figure 3a), except for the sample subset obtained with an NH value of 0.9 and choosing 1 sample per group. However, the sample selection developed with an NH value of 0.9 and choosing √n samples per group produced SEP values really acceptable from 44% of the samples.

Similarly, HC sample selections produce SEP values quite acceptable from 43% and 32% (for the settings √n samples of 28 groups and 1 sample of 45 groups, respectively) of the samples allocated in the calibration set (Figure 3b).

Therefore, the sample selection procedures tested here show great potential for the reduction of the number of samples required for the calibration set. In this way, the necessary resources for the development of MPLS calibrations can be drastically reduced. Finally, some evidence has been found of greater suitability of the dendrogram method for reducing the number of samples of the calibration set.

### 3.4. Comparison of Sample Selection Methods

NH distance and HC analysis have proven to be powerful tools for reducing the number of samples that must be chemically measured to develop a PLS calibration with useful predictive capacity. These tools can reduce the number of samples up to, at least, 44% and 32% of the samples allocating in the FC set, for NH and HC, respectively, without a high loss in the predictive capacity of the models. However, it is not possible to make a comparison of the performance of the two sample reduction methods evaluated, since the number of samples they select is different. Therefore, a comparative procedure was carried out to determine which of these sample selection methods could select the most representative samples.

The described NH and HC procedures were modified to establish the number of samples rather than the distance between them as the threshold. The thresholds were set at 47 and 71 samples, one third and a half of the samples in the FC set, respectively. For obtaining 47 and 71 sample groups, NH values were respectively set at 0.82 and 0.65. In the case of hierarchical clustering, the D_link_/D_max_ had to be reduced to 0.47 and 0.28% to achieve 47 and 71 groups, respectively. Four new sample sets were constructed (NH-47, NH-71, HC-47 and HC-71) with the most spectrally representative samples of each group created. MPLS regressions were performed using these new sample sets (Table 2).

**Figure 4:**
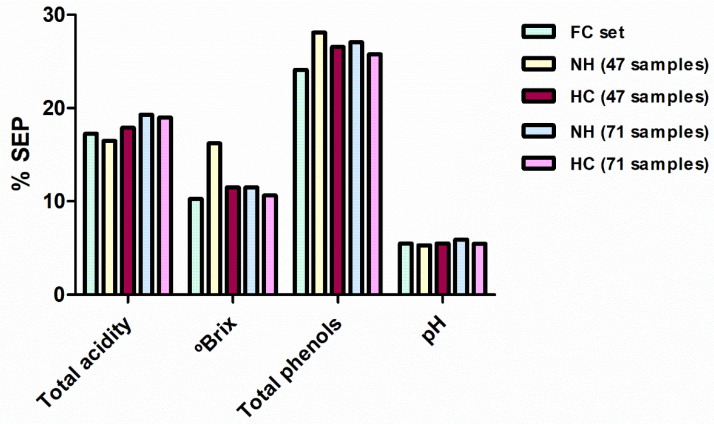
Comparison between the SEP in external validation for the models developed for all the reference parameters for FC, NH-47, HC-47, NH-71 and HC-71 sample sets. SEP are expressed as percentages taking into account the applicability range of their respective models. SEP: standard errors of prediction; NH: Neighbourhood Mahalanobis distance; FC, full calibration; HC: Hierarchical Clustering.

In general, statistics for these new calibrations were slightly inferior to those obtained with the FC set. However, as seen in the previous section, this reduction in performance is offset by the reduced number of samples taken into account (with a consequent reduction in the chemical analyses that would be required). When comparing each developed model with the correspondent FC model using the Fisher test, only the NH-47 selection produced a SEP value for total soluble solid statistically higher in comparison with the FC model. In the remaining models, the differences in the performance (percentage of the SEP with respect to the mean of the range of applicability) of the calibration methods developed with 47 and 71 samples were not clearly found (Figure 4). For both sample selection methods, some reference parameters were better predicted with 47 samples and others with 71. Therefore, it seems that a spectral selection of one-third of the samples (47 samples) can be enough for the adequate prediction of most of the reference parameters. Finally, taking into account the different selection methods applied for constructing these sample sets, HC sample selections produced slightly better SEP values, i.e., six equations developed using HC sets shown better predictions in external validation. However, the differences between the two sample selection methods are so small that both can be considered a good alternative when a reduction in the number of samples is required.

## 4. Conclusions

The results obtained in the present study showed that it is possible to reduce the number of hyperspectral images needed to perform NIR calibrations in grape samples without a significant loss in the predictive capacity. To reduce the number of hyperspectral samples, different sample selection procedures have been tested. Neighbourhood Mahalanobis distance and hierarchical clustering analysis were used to split samples into different groups. Standard errors of prediction (SEP) were calculated for each developed model and compared to those obtained using the full calibration (FC) set. SEPs obtained from the different sample selection sets are comparable to those obtained using the FC set.

Models of high accuracy were obtained with only the 30–40% of the samples belonging to the original entire calibration set. In general, sample subsets obtained from Neighbourhood Mahalanobis distance and from hierarchical clustering analysis produce results with no significant differences to each other, indicating the suitability of both methods to be applied when a reduction of the sample set is required.

## Figures and Tables

**Table 1:** Main statistical parameters for the different Modified Partial Least Square (MPLS) calibrations. In the standard errors of prediction (SEP) column, asterisks (*) indicate statistically significant differences (α = 0.05%) with the corresponding full calibration (FC) model.

Set	Reference Parameters	Spectral Pretreatments	N ^1^	Toutliers	Min ^2^	Max ^3^	RSQ ^4^	SECV ^5^	SEP ^6^
FC ^7^ [13]	TA ^11^	MSC ^14^ 0,0,1,1	141	7	0	45.06	0.96	2.72	3.89
FC ^7^ [13]	TSS ^12^	MSC ^14^ 1,5,5,1	141	8	0	31.40	0.97	1.23	1.61
FC ^7^ [13]	TSP ^13^	SNV ^15^ 2,5,5,1	141	1	0	16.34	0.77	1.77	1.97
FC ^7^ [13]	pH	MSC ^14^ 2,5,5,1	141	2	2.17	4.29	0.92	0.13	0.18
NH-06-1 ^8^	TA ^11^	MSC ^14^ 0,0,1,1	79	2	0	45.42	0.95	2.92	4.18
NH-06-1 ^8^	TSS ^12^	MSC ^14^ 1,5,5,1	79	4	0	29.86	0.93	1.84	1.68
NH-06-1 ^8^	TSP ^13^	SNV ^15^ 2,5,5,1	79	2	0	16.15	0.60	1.96	2.11
NH-06-1 ^8^	pH	MSC ^14^ 2,5,5,1	79	1	2.24	4.21	0.90	0.15	0.19
NH-06-√n ^8,9^	TA ^11^	MSC ^14^ 0,0,1,1	96	2	0	48.37	0.96	3.01	3.98
NH-06-√n ^8,9^	TSS ^12^	MSC ^14^ 1,5,5,1	96	6	0	31.03	0.96	1.53	1.74
NH-06-√n ^8,9^	TSP ^13^	SNV ^15^ 2,5,5,1	96	0	0	17.11	0.78	1.99	2.32
NH-06-√n ^8,9^	pH	MSC ^14^ 2,5,5,1	96	3	2.17	4.26	0.92	0.13	0.17
NH-09-1 ^8^	TA ^11^	MSC ^14^ 0,0,1,1	42	2	0	45.89	0.93	3.60	4.56
NH-09-1 ^8^	TSS ^12^	MSC ^14^ 1,5,5,1	42	3	0	31.57	0.87	2.24	2.66*
NH-09-1 ^8^	TSP ^13^	SNV ^15^ 2,5,5,1	42	2	0	16.08	0.79	1.58	2.37
NH-09-1 ^8^	pH	MSC ^14^ 2,5,5,1	42	1	2.25	4.29	0.94	0.16	0.18
NH-09-√n ^8,9^	TA ^11^	MSC ^14^ 0,0,1,1	62	0	0	45.95	0.93	3.77	4.36
NH-09-√n ^8,9^	TSS ^12^	MSC ^14^ 1,5,5,1	62	3	0	31.27	0.96	1.56	1.86
NH-09-√n ^8,9^	TSP ^13^	SNV ^15^ 2,5,5,1	62	1	0	17.04	0.58	2.13	2.19
NH-09-√n ^8,9^	pH	MSC ^14^ 2,5,5,1	62	2	2.24	4.24	0.93	0.13	0.17
HC-1-1 ^10^	TA ^11^	MSC ^14^ 0,0,1,1	28	2	0	29.34	0.82	3.68	3.21
HC-1-1 ^10^	TSS ^12^	MSC ^14^ 1,5,5,1	28	2	0	31.65	0.83	2.71	3.14 *
HC-1-1 ^10^	TSP ^13^	SNV ^15^ 2,5,5,1	28	1	0	16.11	0.71	2.32	2.29
HC-1-1 ^10^	pH	MSC ^14^ 2,5,5,1	28	1	2.20	4.25	0.87	0.20	0.19
HC-1-√n ^9,10^	TA ^11^	MSC ^14^ 0,0,1,1	61	5	0	45.94	0.96	3.01	4.37
HC-1-√n ^9,10^	TSS ^12^	MSC ^14^ 1,5,5,1	61	3	0	32.03	0.95	1.87	2.14
HC-1-√n ^9,10^	TSP ^13^	SNV ^15^ 2,5,5,1	61	0	0	16.61	0.72	1.90	2.04
HC-1-√n ^9,10^	pH	MSC ^14^ 2,5,5,1	61	0	2.16	4.29	0.93	0.15	0.18
HC-05-1 ^10^	TA ^11^	MSC ^14^ 0,0,1,1	45	1	0	50.43	0.95	3.69	4.46
HC-05-1 ^10^	TSS ^12^	MSC ^14^ 1,5,5,1	45	1	0	31.24	0.96	1.87	1.61
HC-05-1 ^10^	TSP ^13^	SNV ^15^ 2,5,5,1	45	0	0	16.53	0.77	2.03	2.18
HC-05-1 ^10^	pH	MSC ^14^ 2,5,5,1	45	1	2.12	4.36	0.91	0.16	0.18
HC-05-√n ^9,10^	TA ^11^	MSC ^14^ 0,0,1,1	74	4	0	44.69	0.95	3.04	4.36
HC-05-√n ^9,10^	TSS ^12^	MSC ^14^ 1,5,5,1	74	5	0	31.78	0.97	1.38	2.08
HC-05-√n ^9,10^	TSP ^13^	SNV ^15^ 2,5,5,1	74	3	0	15.66	0.66	1.80	1.91
HC-05-√n ^9,10^	pH	MSC ^14^ 2,5,5,1	74	1	2.17	4.33	0.91	0.14	0.18

^1^ N: number of samples (calibration set); ^2^ Min: Minimum estimate; ^3^ Max: maximum estimate; ^4^ RSQ: coefficient of determination (cross-validation); ^5^ SECV: standard error of cross-validation; ^6^ SEP: standard error of prediction in the external validation; ^7^ FC: full calibration set; ^8^ NH: Neighbourhood Mahalanobis distance; ^9^ √n: square root of the number of samples in a group; ^10^ HC: Hierarchical Clustering; ^11^ TA: total acidity (g L^−1^, expressed as tartaric acid equivalents). ^12^ TSS: total soluble solids (°Brix); ^13^ TSP: total skin phenols (mg g^−1^ of skin grape, expressed as gallic acid equivalents); ^14^ MSC: multiplicative scatter correction; ^15^ SNV: standard normal variate.

**Table 2:** Main statistical parameters for the MPLS calibrations developed for comparing the different sample selection methods. In the SEP column, asterisks (*) indicates statistically significant differences (α = 0.05%) with the corresponding FC model.

Set	Reference Parameters	Spectral Pretreatments	N ^1^	Toutliers	Min ^2^	Max ^3^	RSQ ^4^	SECV ^5^	SEP ^6^
FC ^7^ [13]	TA ^10^	MSC ^13^ 0,0,1,1	141	7	0	45.06	0.96	2.72	3.89
FC ^7^ [13]	TSS ^11^	MSC ^13^ 1,5,5,1	141	8	0	31.40	0.97	1.23	1.61
FC ^7^ [13]	TSP ^12^	SNV ^14^ 2,5,5,1	141	1	0	16.34	0.77	1.77	1.97
FC ^7^ [13]	pH	MSC ^13^ 2,5,5,1	141	2	2.17	4.29	0.92	0.13	0.18
NH-47 ^8^	TA ^10^	MSC ^13^ 0,0,1,1	47	2	0	52.57	0.96	3.68	4.33
NH-47 ^8^	TSS ^11^	MSC ^13^ 1,5,5,1	47	1	0	30.96	0.90	2.52	2.51 *
NH-47 ^8^	TSP ^12^	SNV ^14^ 2,5,5,1	47	3	0	14.98	0.74	1.54	2.10
NH-47 ^8^	pH	MSC ^13^ 2,5,5,1	47	1	2.12	4.34	0.96	0.13	0.17
NH-71 ^8^	TA ^10^	MSC ^13^ 0,0,1,1	71	4	0	45.78	0.97	2.73	4.42
NH-71 ^8^	TSS ^11^	MSC ^13^ 1,5,5,1	71	5	0	30.60	0.93	1.89	1.76
NH-71 ^8^	TSP ^12^	SNV ^14^ 2,5,5,1	71	3	0	15.74	0.66	1.72	2.13
NH-71 ^8^	pH	MSC ^13^ 2,5,5,1	71	2	2.26	4.24	0.90	0.13	0.19
HC-47 ^9^	TA ^10^	MSC ^13^ 0,0,1,1	47	1	0	49.51	0.95	3.47	4.43
HC-47 ^9^	TSS ^11^	MSC ^13^ 1,5,5,1	47	2	0	30.50	0.94	1.71	1.76
HC-47 ^9^	TSP ^12^	SNV ^14^ 2,5,5,1	47	1	0	16.50	0.78	1.87	2.19
HC-47 ^9^	pH	MSC ^13^ 2,5,5,1	47	4	2.12	4.38	0.95	0.12	0.18
HC-71 ^9^	TA ^10^	MSC ^13^ 0,0,1,1	71	1	0	47.90	0.93	3.87	4.55
HC-71 ^9^	TSS ^11^	MSC ^13^ 1,5,5,1	71	5	0	31.42	0.96	1.59	1.67
HC-71 ^9^	TSP ^12^	SNV ^14^ 2,5,5,1	71	0	0	16.11	0.59	2.02	2.08
HC-71 ^9^	pH	MSC ^13^ 2,5,5,1	71	2	2.19	4.30	0.93	0.13	0.18

^1^ N: number of samples (calibration set); ^2^ Min: Minimum estimate; ^3^ Max: maximum estimate; ^4^ RSQ: coefficient of determination (cross-validation); ^5^ SECV: standard error of cross-validation; ^6^ SEP: standard error of prediction in the external validation; ^7^ FC: full calibration set; ^8^ NH: Neighbourhood Mahalanobis distance; ^9^ HC: Hierarchical Clustering; ^10^ TA: total acidity (g L^−1^, expressed as tartaric acid equivalents). ^11^ TSS: total soluble solids (°Brix); ^12^ TSP: total skin phenols (mg g^−1^ of skin grape, expressed as gallic acid equivalents) ); ^13^ MSC: multiplicative scatter correction; ^14^ SNV: standard normal variate.

## Data Availability

Data is contained within the article.

## Supplementary Materials

The following are available online at https://www.mdpi.com/2304-8158/10/2/233/s1. Figure S1: Example spectra of the red and white grape samples in the NIR zone between 950 and 1650 nm.
