# Supplementary material for: Reduction of the Number of Samples for Cost-Effective Hyperspectral Grape Quality Predictive Models
Source: Foods. 2021 Jan 23;10(2):233. doi: 10.3390/foods10020233 (PMC7912666; doi:10.3390/foods10020233)
Supplement: Supplementary file 1 [file foods-10-00233-s001.pdf]

foods

Supplementary material

Reduction of the Number of Samples for Cost-Effective Hyperspectral Grape Quality Predictive Models

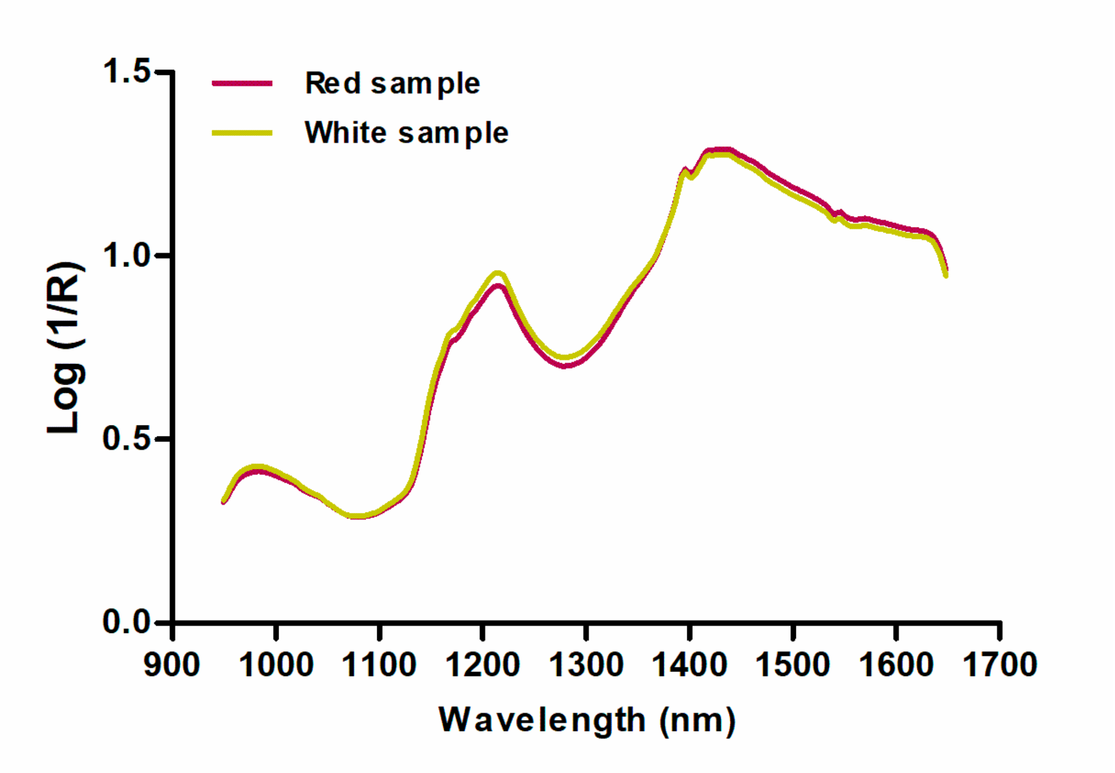

Figure S1. Example spectra of the red and white grape samples in the NIR zone between 950 and 1650 nm. R: reflectance.
